# Herbicide glufosinate inhibits yeast growth and extends longevity during wine fermentation

**DOI:** 10.1038/s41598-017-12794-6

**Published:** 2017-09-29

**Authors:** Beatriz Vallejo, Cecilia Picazo, Helena Orozco, Emilia Matallana, Agustín Aranda

**Affiliations:** 1 Institute of Agrochemistry and Food Technology (IATA-CSIC), Paterna, Valencia Spain; 2 Department of Biochemistry and Molecular Biology, University of Valencia, Burjassot, Valencia Spain; 3 Institute for Integrative Systems Biology (I2SysBio), University of Valencia-CSIC, Paterna, Valencia Spain

## Abstract

Glufosinate ammonium (GA) is a widely used herbicide that inhibits glutamine synthetase. This inhibition leads to internal amino acid starvation which, in turn, causes the activation of different nutrient sensing pathways. GA also inhibits the enzyme of the yeast *Saccharomyces cerevisiae* in such a way that, although it is not used as a fungicide, it may alter yeast performance in industrial processes like winemaking. We describe herein how GA indeed inhibits the yeast growth of a wine strain during the fermentation of grape juice. In turn, GA extends longevity in a variety of growth media. The biochemical analysis indicates that GA partially inhibits the nutrient sensing TORC1 pathway, which may explain these phenotypes. The *GCN2* kinase mutant is hypersensitive to GA. Hence the control of translation and amino acid biosynthesis is required to also deal with the damaging effects of this pesticide. A global metabolomics analysis under winemaking conditions indicated that an increase in amino acid and in polyamines occurred. In conclusion, GA affects many different biochemical processes during winemaking, which provides us with some insights into both the effect of this herbicide on yeast physiology and into the relevance of the metabolic step for connecting nitrogen and carbon metabolism.

## Introduction

Wine yeast faces many adverse conditions when grape juice fermentation starts^1^. High sugar concentration, low pH and oxygen are the stress conditions naturally imposed to yeasts. Regarding artificial challenges, the addition of sulfite as a preservative and antioxidant is a widely extended enological practice to which wine strains of the yeast *Saccharomyces cerevisiae* have well adapted through genetic changes that affect sulfite efflux^2^. Besides, the effect of the chemical compounds that may be present in grape juice and grape skins due to vine agronomic practices has been less well studied and remains poorly understood. The effect on growth, fermentative capacity and/or longevity of pesticides like herbicides, insecticides and fungicides may differ in distinct winemaking areas and years. The concentration of most fungicides lowers during fermentation and has no impact on wine fermentation^3^, although many pesticides remain throughout the vinification process^4^. For instance, folpet is the most widely found pesticide in grape juice (in approximately 14.5% of samples around the world)^5^, but is degraded by photolysis and hydrolysis during fermentation^6^. Although most pesticides do not impact yeast performance during fermentation, their presence in the field definitively influences the yeast flora on grape surfaces^7^, which may influence the following spontaneous fermentation. This could also be the case of some herbicides that affect yeast metabolism. In some cases the commercial version of the herbicide has more deleterious effects than the pure active ingredient^8^.

Apart from having a global effect on yeast performance, some pesticides may have specific effects that derive from their mechanism of action, and could be used as an interesting tool to understand yeast physiology under industrial conditions. For instance, 3-Amino-1,2,4-triazole (3-AT) is the component of herbicide amitrol and, thanks to its inhibition of histidine biosynthesis, it has been widely used in research to study the mechanisms of amino acid starvation. It also has a potential impact on most grape fungal microbiota^9^. The best characterized pathway that controls amino acid biosynthesis is the General Amino Acid Control (GAAC) system^10^. When amino acids are scarce or their biosynthesis is blocked by inhibitors such as 3-AT, empty tRNAs are sensed by Gcn2 kinase, which blocks general translation initiation, but promotes the translation of transcription factor Gcn4 that activates the biosynthesis of all proteinogenic amino acids. A similar effect is caused by the herbicides of the family of the sulfonylureas, e.g., sulfometuron methyl (SM), which inhibit acetolactate synthase, required for leucine, isoleucine and valine biosynthesis^11,12^. The mutants in Gcn2 kinase then become hypersensitive to these inhibitors^13,14^. Global analyses indicate that 3-AT and SM increase the amino acid biosynthetic genes in a Gcn4-dependent way^11,15^. Other molecules that inhibit amino acid biosynthesis affect metabolism through other pathways. Methionine sulfoximine (MSX) blocks glutamine synthase (GS), a central reaction in nitrogen assimilation in yeast that synthesizes glutamine from glutamic acid and free ammonium. This glutamine starvation triggers the inhibition of the Target Of Rapamycin (TOR) kinase^16^. The TOR pathway controls nitrogen catabolite repression (NCR), which promotes the use of alternative poor nitrogen sources when rich sources like ammonia or glutamine are deprived^17^. Both the GAAC and TOR pathways are interrelated and modulated by the pathway sensing carbon source, such as Protein Kinase A and Snf1^18^. Nutrient shortage, known as dietary restriction, extends chronological life span (i.e., the time in which cells are viable in the stationary phase^19^). This phenomenon is controlled by the nutrient-sensing pathways in which the TOR pathway is clearly relevant, and also under winemaking conditions^20,21^. Therefore, such amino acid biosynthesis is able to strongly influence yeast physiology.

Glufosinate ammonium (GA, also called phosphinothricin) is a wide-spectrum herbicide that reversibly inhibits glutamine synthase (Gln1 in yeast) due to its similarity to glutamate^22^. This leads to ammonia accumulation, which eventually disrupts photosynthesis in plants. Its effect on the yeast *S. cerevisiae* is less understood. Addition of GA causes cell wall defects in laboratory strains similar to *GLN1* deletion, and cells become round and enlarged^23^. In this paper we studied the effect of GA on wine yeast under winemaking conditions. By using mutations on amino acid-sensing pathways, we wished to attempt to establish its mechanism of action and its use as a tool to study yeast metabolism. GA affects more than one nutrient-sensing pathway and also has effects on yeast longevity, both of which suggesting additional targets yet to be described. A metabolomics analysis that gives us hints on the interconnection between carbon and nitrogen metabolism in wine yeast has been performed.

## Results

### Impact of glufosinate ammonium on grape juice fermentation

**Figure 1:**
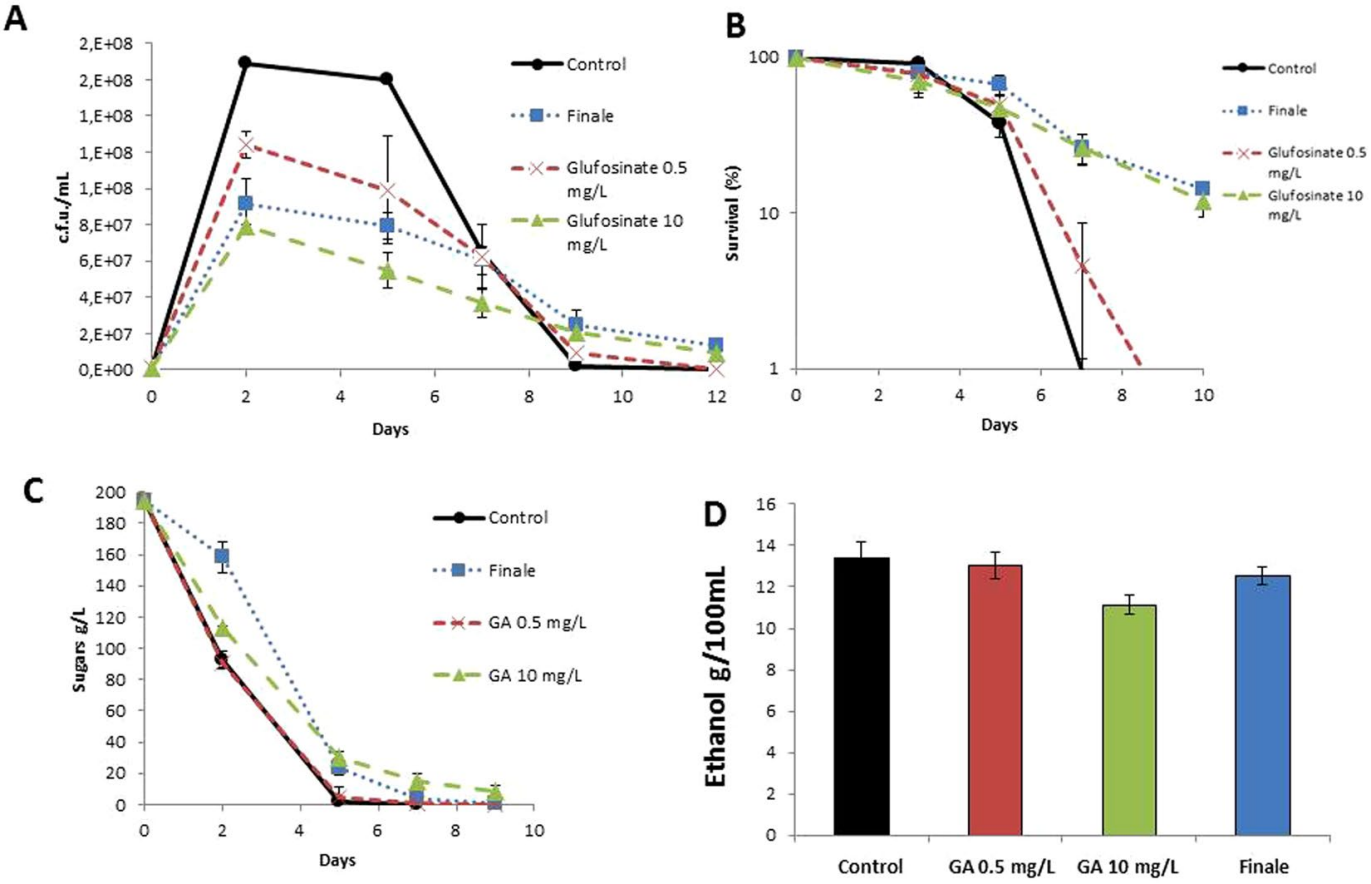
Glufosinate ammonium impacts growth and longevity in winemaking conditions. (**A**) Viable cell number in natural grape juice fermentations of the commercial EC1118 wine strain with different amounts of GA, measured as cfu/mL. (**B**) Survival curve along the aforementioned vinifications. Viable cell number at day 2 from Panel A was taken as 100% survival. (**C**) Sugar consumption during fermentations. (**D**) Ethanol (at the end of fermentations). Experiments were done in triplicate. The mean and standard deviation are provided.

In order to assess the impact of GA on winemaking, we first tested the effect on the fermentation of natural red grape juice by the widely used commercial *S. cerevisiae* strain EC1118. At the time of inoculation, we added two different amounts of pure GA (Sigma Inc.). We added 10 mg/L as a reference because this amount has proved to affect growth and to damage cell walls under laboratory conditions^23^. It was tested at a lower dose of 0.5 mg/L. That was similar to the maximum residue levels (mg/kg) allowed by the EU for wine grape up to 2015. In 2016 it was lowered to 0.15 mg/kg. Figure 1A shows the growth profile of such fermentations. The higher GA concentration had a profound impact on cell growth. Maximum cell counts lowered by half after 2 days of growth, when the cell count peaked under the control conditions. Even the smaller amount of herbicide caused reduced growth, but to a lesser extent. Therefore, glutamine synthetase activity is required for yeast growth in grape must, even though this amino acid is present in the growth medium^24^. Finale (Bayer CropScience, S.L.), a commercial herbicide that contained 15% GA was also tested to render a final 10 mg/L concentration of the active compound (Fig. 1A). The effect on growth was also strong, similar to the pure chemical at a similar concentration but slightly reduced. This suggests that the amount of active ingredient was slightly lower than expected, probably due to the lower degree of purity of the manufacturing process or its storage. However, the effect of this herbicide on cell proliferation was strong.

After reaching their maximum cell densities, yeasts started to die. By taking cell counts on day 2 as 100% viability, we obtained a chronological aging profile (Fig. 1B). EC1118 is a strain with a relatively high aging rate in both laboratory media and grape juice fermentation^25^, as shown by the sharp decline in viability (Fig. 1B). Addition of 0.5 mg/L of GA slightly extended longevity, but this effect was clearer when pure GA was used at 10 mg/L or at the same concentration but in the form of Finale. Therefore, GS inhibition by GA extended longevity under winemaking conditions, as expected for TOR inhibition^20^. Regarding sugar fermentation, the smaller amount of GA did not significantly influence the consumption of reducing sugars (Fig. 1C), which finished around 5 days as it did under the control condition. The higher GA dose lowered the sugar metabolism rate, particularly when pure, and that may contribute to extend lifespan. This may also reflect the lower cell density imposed by the herbicide. Ethanol was similar in all cases (Fig. 1D), but slightly lower when 10 mg/L of pure GA was used, which might reflect the slower sugar consumption rate. Therefore, GA impacts growth, longevity and sugar consumption during grape juice fermentation.

### Genetic determinants of glufosinate ammonium sensitivity

**Figure 2:**
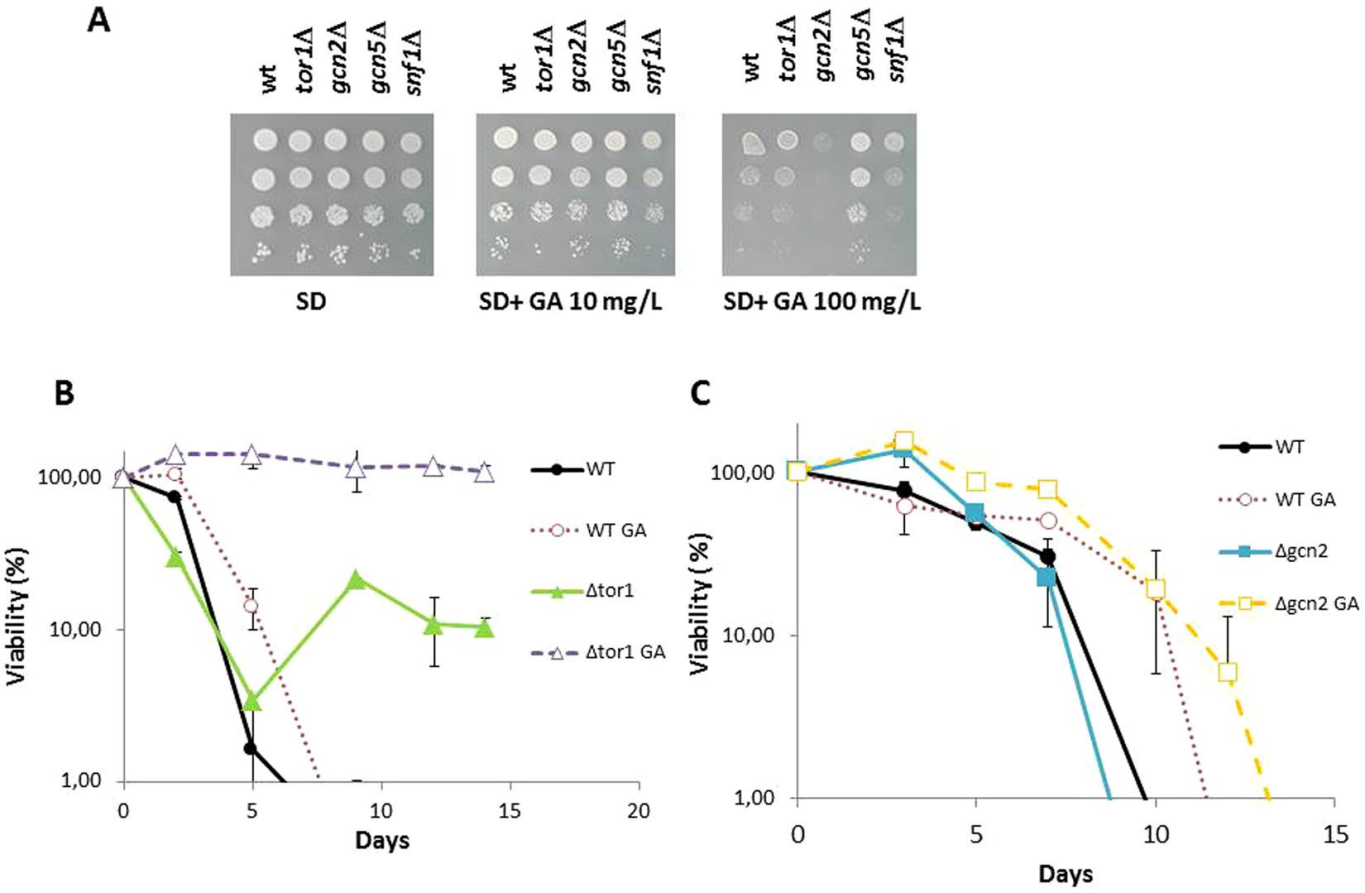
Deletion of kinase Gcn2 impairs tolerance to glufosinate ammonium. (**A**) Spot assays of wine yeast C9 mutants in nutrient sensing pathways on SD plates containing GA. (**B**) Standard chronological life span analysis in SC medium of *tor1*Δ mutant in the presence and absence of 10 mg/L GA. (**C**) Same as panel B) for *gcn2*Δ mutant. Experiments were done in triplicate.

After testing the effect of GA on a commercial strain in grape juice, the next set of experiments was carried out in haploid strain C9, a derivative of commercial wine strain L2056^26^. This strain allows easy genetic manipulation, and past experiments have shown that it reflects what happens in diploid commercial strains^20,21^. A fermentation of this C9 strain in natural grape juice indicated that GA also cause a reduction of growth (Supplementary Figure S1A) in a similar way that was seen for EC1118 strain (Fig. 1A). Spot analysis of both wine yeasts on plates containing GA together with two laboratory strains (Supplementary Figure S1B) enable a direct comparison. Although there are subtle differences between genetic backgrounds, wine yeasts do no perform differently form laboratory strains against this herbicide, so we carry on with genetic manipulation of wine strain C9, as laboratory strains do not perform well on winemaking conditions. The mutants in the most relevant pathways for nitrogen signaling, such as *gcn2*Δ from GAAC and *tor1*Δ from the TOR pathway, were tested. An important mutant for glucose repression such as *snf1*Δ.was included as a mutation control in other signaling pathways, and the transcriptional regulator of stress response *gcn5*Δ.was also included. Sensitivity to GA was tested on SD minimal plates without amino acids, a medium where amino acid starvation renders growth defects. These mutants showed no significant growth defect when spotted on the minimal medium SD plates that contained 10 mg/L GA (Fig. 2A). However, differences in growth emerged when the concentration was raised 10-fold. The mutation of chromatin control factor *GCN5* showed a slight increase in tolerance, the mutation in *GCN2* kinase caused the most striking growth defect, while the mutations in the *TOR1* and *SNF1* pathways rendered no evident phenotype. In relation to these conditions, we cannot rule out effects on the TOR pathway as the *TOR2* gene may give enough TOR complex to deal with this inhibition.

We then studied the impact of mutation in nitrogen-sensing pathways on growth and aging in different environments. In order to determine the role of GS inhibition on yeast aging, chronological life span experiments were carried out in the standard way and in complete synthetic medium SC following the drop in viability after 3 days (Fig. 2B). This medium enabled us to compare these results with previous experiments^21,27^. 10 mg/L GA caused CLS extension in this medium. As described in the laboratory strains, *TOR1* deletion in wine yeasts extended CLS. Therefore, the interventions that blocked nutrient-sensing pathways also extended longevity in yeast wine strains. Addition of GA to the *tor1*Δ mutant led to an additional life span extension, which implies that it may act through a parallel pathway, at least partially. *GCN2* deletion did not bring about any significant change in longevity under these conditions, and addition of GA equally extended CLS in the *gcn2*Δ mutant (Fig. 2C). What this indicates is that Gcn2 is not as relevant in a medium rich in amino acids (Fig. 2C) than in a medium without amino acids (Fig. 2A).

**Figure 3:**
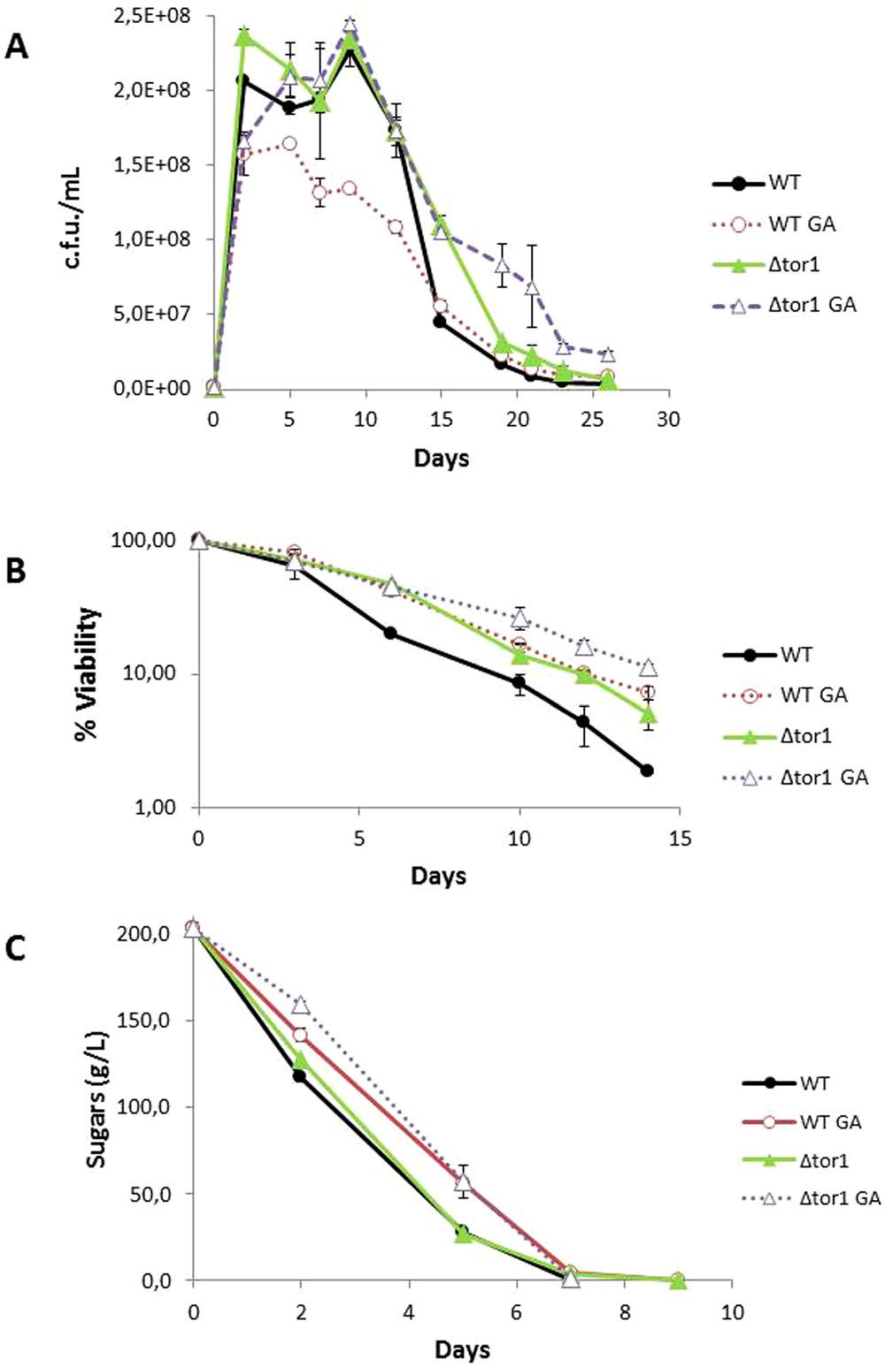
Glufosinate interacts with *TOR1* to affect growth and longevity but no sugar consumption in winemaking conditions. (**A**) Viable cell number in synthetic grape juice fermentations of the C9 wine strain and its *tor1*Δ derivative, with or without 10 mg/L GA, measured as cfu/mL. (**B**) Survival curve along the aforementioned vinifications. Viable cell number at day 5 from Panel A was taken as 100% survival. (**C**) Sugar consumption during fermentations. Experiments were done in triplicate.

Next the effect of deletions *TOR1* and *GCN2* on GA sensitivity was tested under winemaking conditions. From this point onward, a synthetic grape juice was used to compare with previous results by our and other laboratories^20,21,28^ and to perform global analyses in a more reproducible medium (see below). The growth profile of the fermentation of parental strain C9 in the presence of GA 10 mg/L (Fig. 3A) was similar to that observed in natural grape juice (Supplementary Figure S2A). Growth was reduced and the culture had lower cell density. The survival plot (Fig. 3B) shows a CLS extension, as observed in natural grape juice (Fig. 1B). Therefore, the effect of GA on synthetic grape juice was similar to that observed in real grape juice. *TOR1* deletion did not impair cell growth (Fig. 3A), but extended CLS, which occurred in the laboratory media (Fig. 3B). Surprisingly, *TOR1* deletion partially suppressed the defect of growth caused by GA. The combination of both achieved full cell density, but at later fermentation times. As seen in the previous CLS experiments, addition of GA further extended the CLS extension caused by *TOR1* deletion (Fig. 3B). Therefore, GA affected other longevity mechanisms apart from TOR pathway inhibition. *TOR1* deletion had no impact on sugar consumption (Fig. 3C) and GA delayed this consumption, regardless of the genetic background. Thus both interventions were not additive.

**Figure 4:**
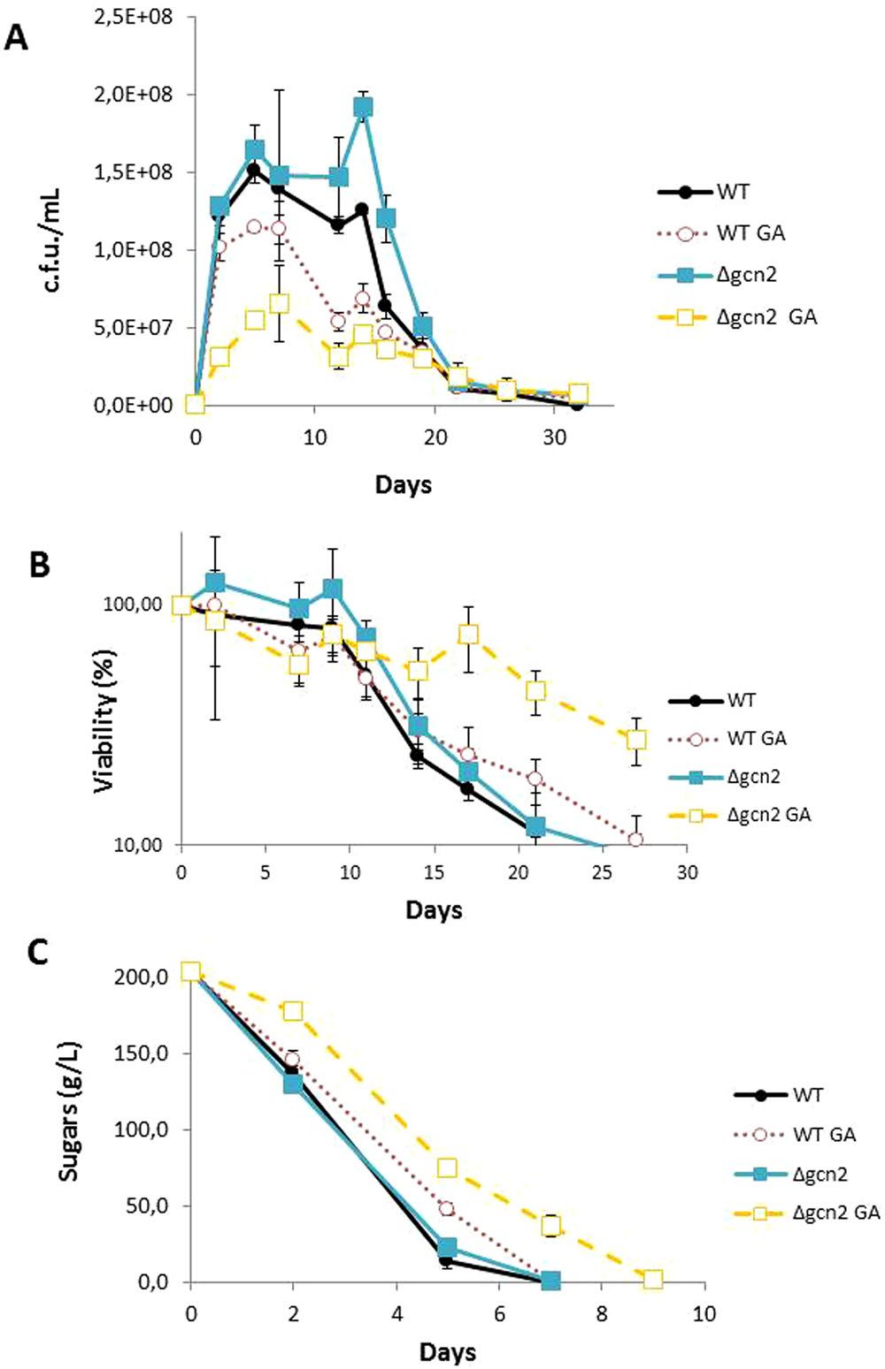
Deletion of *GCN2* increases sensitivity to glufosinate in winemaking conditions. (**A**) Viable cell number in synthetic grape juice fermentations of the C9 wine strain and its *gcn2*Δ derivative with or without 10 mg/L GA. (**B**) Survival curve of the aforementioned vinifications. (**C**) Sugar consumption during fermentations. Experiments were done in triplicate.

Under the same conditions, *GCN*2 deletion displayed no growth defect. In fact cells reached higher viability at later times (Fig. 4A), which rendered extended early longevity, although maximal longevity was similar (Fig. 4B). As grape juice is a rich medium, it seems clear that the systems which control amino acid biosynthesis are less relevant than those devoted to catabolism. Addition of GA led to strongly repressed growth, similarly to that observed on plates (Fig. 2A). CLS extension was bigger when GA was added (Fig. 4B), which was also observed for *TOR1*. Hence, there was also an additive effect. *GCN5* deletion did not affect growth under winemaking conditions, but once again showed a strong effect in combination with GA (Supplementary Figure S2A). This was not observed in the assays that used plates (Fig. 2A), and suggests that the involvement of the SAGA complex in dealing with GA is environment-dependent, and more relevant under fermentation conditions. The effect on CLS was once again additive (Supplementary Figure S2B). Regarding sugar consumption, *GCN2* deletion had no impact on sugar metabolism, but *GCN5* deletion did (Fig. 4C), while GA caused a delay in fermentation in all cases.

### Partial TOR repression by GA during fermentation

**Figure 5:**
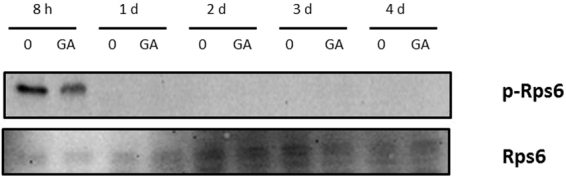
GA partially inhibits TORC1 at the beginning of fermentation. Western blot of proteins from a fermentation in synthetic grape juice of strain C9 with or without 10 mg/L GA. Phosphorylated and total Rsp6 are indicated. Experiments were performed twice with similar results and one set is shown.

In order to assess any direct impact of GA on TOR activity, a pathway known to be regulated by glutamine synthetase inhibition^16^, a specific assay was performed (Fig. 5). The C9 strain was grown in synthetic grape juice MS300, with or without 10 mg/L GA, and samples were taken at different times. A target of TOR activity is small ribosomal protein Rps6, whose phosphorylation state disappears after TOR inhibition^29,30^. The phosphorylated state was followed with a specific anti-phospho Rps6. This protein is only phosphorylated during the first hours of fermentation. After 1 day the protein is dephosphorylated, an event related to nitrogen starvation. The overall result in the fermentation that contained GA was similar. However, the initial phosphorylation levels were lower, which suggests that the herbicide caused the partial inhibition of TOR activity, which could explain the delay in growth caused by it.

### Glufosinate global impact on metabolism during winemaking

**Figure 6:**
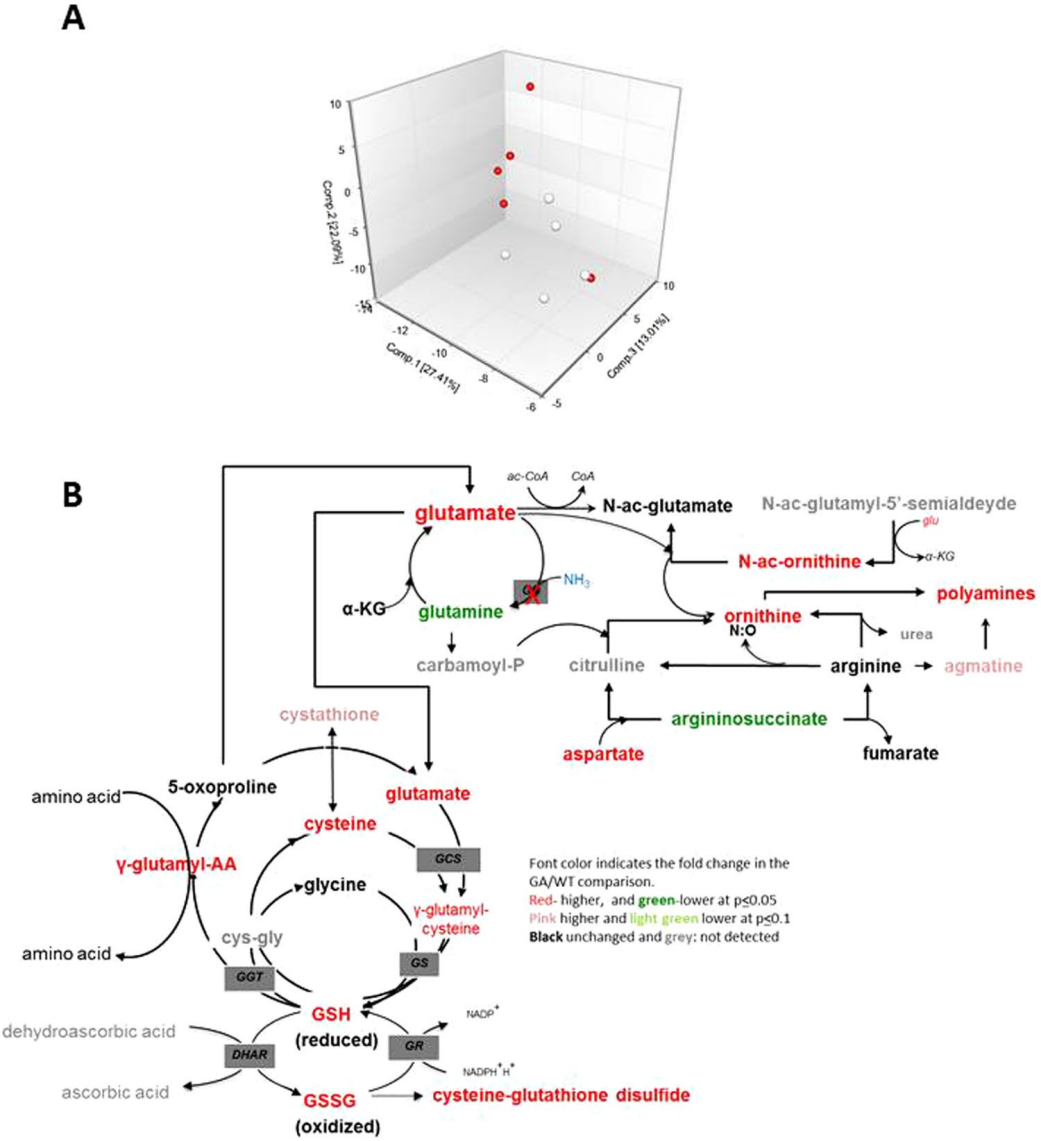
GA impacts many aspects of the metabolome during winemaking (**A**) PCA analysis of the five samples of the control and GA-treated cells grown in synthetic must to the stationary phase. (**B**) Metabolites in steps linked to the glutamine synthetase reaction inhibited by GA. Green indicates down-regulation and red indicates up-regulation.

As the control of nutrient-sensing pathways does not fully explain the impact of GA on wine yeast fermentation, a global metabolomics analysis was carried out to obtain the full picture of the impact of this chemical on metabolism. The C9 strain was grown in synthetic grape juice, with and without GA 10 mg/L, and five samples were taken in the stationary phase when sugars were around 50 g/L. Of all the 423 identified metabolites, 180 were changed by treatment (with a p ≤ 0.05), 97 were up-regulated and 83 were down-regulated with 1.2 fold change (See Supplementary Table S1). A principal component analysis (PCA) showed that the biochemical profiles of the two groups examined herein were distinct (Fig. 6A). As expected, glutamine and asparagine, the main products of nitrogen assimilation and storage, were lower in the GA-treated cells, which confirms that the expected mode of action of GA acts under these conditions. However, most other proteinogenic amino acids accumulated in the GA-treated cells compared to the control, most likely due to reduced catabolism, as many amino acid and purine catabolic products were reduced. That could explain the negative effect of GA on growth. This result suggests adaptation in the GA-treated yeast cells to minimize the reactions that would produce ammonia, which would prove toxic due to the impaired assimilation machinery. Glutamate, the substrate for GS, appeared to divert into the acetyl cycle for ornithine production (Fig. 6B). Furthermore, higher levels of polyamine putrescine and its catabolite acetyl-putrescine, products of ornithine, were also observed in the GA-treated cells compared to the untreated condition. A rise in polyamines could contribute to extended longevity as these molecules have a pro-longevity effect^31^. Glutamate did not appear to be directed into amino acid biosynthesis because the glutamate-derived amino acids proline, arginine and γ-aminobutyrate (GABA) remained unchanged. Most of the compounds associated with the glutathione cycle detected in this study were high compared to the reference condition. This suggests high levels of oxidative stress following GA treatment, although no increase was observed in hydroxy-fatty acids, which are generally reported to be produced during oxidative stress by membrane peroxidation. Alternatively, intrinsically better antioxidant defense may explain extended longevity.

One of the most dramatic changes in this data set was the 50-fold higher levels of xanthosine observed in the GA-treated line compared to the reference condition. Xanthine and xanthosine are purine salvage products that may be further converted into urate for degradation. However, this conversion into urate occurs with the release of NH_3_. It is quite likely that such high xanthosine levels, which accumulated in the GA-treated cells to prevent ammonia production, can be toxic to cells if GS activity is absent. Another significant observation was the 123-fold accumulation of nicotinic acid adenine dinucleotide (NAAD) in the GA-treated cells compared with the WT. NAAD converts into NAD by the action of NAD synthase, which uses glutamine as a nitrogen donor. This result suggests that glutamine cannot be replaced with any other nitrogen donor in this reaction, which causes a lesion in NAD production in response to GA treatment. This also explains the increment in NAAD precursors on the NAD salvation pathway, like nicotinic acid mononucleotide (NaMN), nicotinic acid and nicotinamide (Supplementary Figure S3). Therefore, GA affects many pathways merely by its inhibition of ammonium assimilation, which might have a strong impact on yeast physiology and might influence growth and longevity.

## Discussion

This paper explores the impact of herbicide glufosinate ammonium on wine yeast performance and its ways of action. Even small amounts of this compound caused a delay in growth during grape juice fermentation (Fig. 1). It also extended longevity in a variety of environments. Therefore, this herbicide strongly impacts yeast physiology, and was used as a good tool to study the relevance of the affected metabolic reaction, the inhibition of glutamine synthetase (GS), a key step is ammonium assimilation, which, together with the glutamate synthase reaction, connected carbon and nitrogen metabolisms. GS inhibition by another amino acid analog, methionine sulfoximine (MSX), inhibited the TOR pathway, which was also the case with GA during winemaking (Fig. 5). The observed inhibition was partial, but it is worth noting that MSX is normally used at 2 mM, while GA was used at 0.05 mM in this experiment. However, it is clear that TOR inhibition is not the only way in which GA affects yeast as *TOR1* deletion had an additive effect on addition of GA in chronological life span extension terms. Another pathway related to TOR in terms of response to nutrient starvation is GAAC. Indeed this pathway is related to GA response as *GCN2* deletion showed hypersensitivity to high GA doses (Fig. 2). Similarly, *GCN2* deletion is sensitive to other molecules that inhibit amino acid synthesis, like amitrol^32^ or sulfometuron methyl^13^. The metabolomics analysis indicated that most proteinogenic amino acids were up-regulated by addition of GA (Supplementary Table S1), besides those that derived directly from glutamine. This may indicate that GA triggers a general synthesis of amino acids, and that this mechanism may require the action of Gcn2 kinase, which senses amino acid starvation. However according to the metabolomics analysis, many amino acid and purine catabolic products were reduced after GA treatment. Hence other mechanisms that regulate catabolic processes may also be triggered by the herbicide. When GA treatment was compared with the deletion of kinase Sch9 (a component of the TOR pathway) and the overexpression of transcription factor Gcn4 was done under the same conditions^33^, many metabolites (229 and 244, respectively) changed, and even more so than those that changed when untreated cells as were taken as a reference (180). Therefore, GA seems to have a different impact from the alteration to a single signaling pathway.

Although cells increased most amino acids after GA treatment, inhibition of both TOR and GAAC could have a negative effect on translation, which might be a potential cause of the growth defect caused by the herbicide. The negative impact on purines could also contribute to difficult gene expression. The metabolomic analysis indicated no impact on glycolysis. The reduction in sugar consumption due to GA addition (Figs 3 and 4) may reflect a general loss in fitness rather than a direct effect on carbohydrate catabolism.

Chronological life span extension may be related to several metabolic changes. Accumulation of polyamines due to diversion from excess glutamate may contribute to it as these molecules are known as pro-longevity factors^31^. Although glutamine is required for the synthesis of NAD, NAD^+^ only slightly reduces, while its reduced version NADH is overaccumulated by addition of GA. This shift may influence a well-known aging regulator that uses NAD^+^ as a substrate, sirtuin Sir2. In a previous work, we showed that Sir2 is detrimental for longevity under wine-making conditions, while it is required for laboratory conditions^20^. The fact that GA influences longevity under different conditions may rule out this possibility. Besides, partial TOR inhibition may contribute to CLS extension by other mechanisms.

## Methods

### Yeast strains and growth media

Commercial wine strain EC1118 was obtained from Lallemand Inc. (Montreal, Canada), while haploid wine strain C9 (*Mat* a, *ho*::*loxP*) was a gift from Michelle Walker^26^. The *TOR1* and *GCN5* gene deletions in C9 were previously performed^21^. To perform the *GCN2* and *SNF1* gene disruptions, the recyclable selection marker *loxP*-*kanMX*-*loxP* from plasmid pUG6^34^ was used. Yeast transformations were done by the lithium acetate method^35^.

Rich YPD medium (1% yeast extract, 2% bactopeptone, 2% glucose) and complete minimal SC medium, which contained 0.17% yeast nitrogen base, 0.5% ammonium sulfate, 2% glucose and 0.2% drop-out mix with all the amino acids, was used to propagate yeasts and for chronological life span experiments, respectively^36^. SD is equal to SC without the mix of amino acids. The solid plates contained 2% agar, and 20 μg mL^−1^ geneticin if required. Red grape juice (Bobal variety) was a gift from Bodegas Murviedro (Requena, Spain). It was sterilized overnight with 500 μg/L of dimethyl dicarbonate. Synthetic grape juice MS300 was made as previously described^37,38^.

### Grape juice fermentations and chronological life span experiments

For the microvinification experiments in both natural and synthetic grape juices, cells from the 2-day cultures in YPD were inoculated at a final concentration of 10^6^ cells/mL in filled-in conical centrifuge tubes with 30 mL of medium. Incubation was done with very low shaking at 25 °C. Vinification progress was followed by determining viable cell number (by serial dilution, plating and colony counts) and sugar consumption, as previously described^20^. The reducing sugars during fermentation were measured by the reaction to DNS (dinitro-3,5-salycilic acid)^39^. Ethanol was measured with the kits provided by r-Biopharm following the manufacturer’s instructions. Survival plots were drawn by taking the highest viable cell number point (around 2–5 days) as 100% survival.

The CLS experiments were carried out under laboratory conditions in the standard way^40^. Precultures were grown overnight on YPD and were then inoculated in synthetic complete SC media at an OD_600_ of 0.1. After 3 days of growth at 30 °C, aliquots were taken, diluted and plated. Colonies were counted and the percentage of survival was calculated by taking day 3 of growth as 100% survival.

### Western blotting

Cells from vinifications were taken and frozen to be broken afterwards with one volume of glass beads in a buffer that contained Tris-HCl 0.1 M, pH 7.5, NaCl 0.5 M, MgCl_2_ 0.1 M, NP40 1% (v/v), PMSF 10 mM and protease inhibitors (complete Mini, EDTA-free from Roche) and phosphatase inhibitors^20^. Extracts were diluted after quantification by the Bradford method (Biorad Inc. Hercules, CA, USA) in loading buffer for SDS-PAGE. After electrophoresis in an Invitrogen mini-gel device, the gel was blotted onto PVDF membranes for Western blot analysis with a Novex semy dry blotter (Invitrogen, Carlsbad, CA, USA). The anti-phospho-S6 ribosomal protein antibody was obtained from Cell Signalling Technology (Beverly, MA, USA) and the anti-Rps6 antibody was acquired from Abcam (Cambridge, MA, USA). The ECL Western blotting detection system (GE) was used following the manufacturer´s instructions.

### Metabolomic analysis

Five cultures of C9 with and without 10 mg/L GA were grown in MS300 synthetic grape juice were taken in the stationary phase, when remaining sugars were around 50 g/l. Cell pellets were frozen and analyzed by Metabolon Inc. (Durham, NC, USA). Samples were prepared in the MicroLab STAR system (Hamilton Company). The resulting extract was divided into four fractions: two for the analysis by two separate reverse phase (RP)/UPLC-MS/MS methods with positive ion mode electrospray ionisation (ESI), one for the analysis by RP/UPLC-MS/MS with the negative ion mode ESI, and one for the analysis by HILIC/UPLC-MS/MS with the negative ion mode ESI. The informatics system consisted of four major components: the Laboratory Information Management System (LIMS), the data extraction and peak-identification software, the data processing tools for QC and compound identification, and a collection of information interpretation and visualization tools used by data analysts. A principal components analysis was performed. Each principal component is a linear combination of every metabolite, and principal components are uncorrelated. The number of principal components equals the number of observations. The first principal component is computed by determining the coefficients of the metabolites that maximize the variance of the linear combination. The second component finds the coefficients that maximize variance with the condition that the second component is orthogonal to the first. The third component is orthogonal to the first two components, and so on.

### Data Availability

All data generated or analysed during this study are included in this published article (and its Supplementary Information files).

## Electronic supplementary material

Supplementary Information

Supplementary Table S1
