# Supplementary material for: Herbicide glufosinate inhibits yeast growth and extends longevity during wine fermentation
Source: Sci Rep. 2017 Sep 29;7:12414. doi: 10.1038/s41598-017-12794-6 (PMC5622092; doi:10.1038/s41598-017-12794-6)

Supplementary Information for:

Herbicide glufosinate inhibits yeast growth and extends longevity during wine fermentation

Beatriz Vallejo[1,2,3], Cecilia Picazo[1,2,3], , Helena Orozco[1,2,3] , Emilia Matallana[1,2,3] and Agustín Aranda[1,3]

[1.] Institute of Agrochemistry and Food Technology (IATA-CSIC), Paterna, Valencia, Spain. [2.] Department of Biochemistry and Molecular Biology, University of Valencia, Burjassot, Valencia, Spain. [3.] Institute for Integrative Systems Biology (I2SysBio), University of Valencia-CSIC, Paterna, Valencia, Spain.

This file contains 3 Supplementary Figures and the original Western blot images used to preparare Figure 5

Supplementary Figure S1. Sensitivity to GA in different genetic backgrounds. A) Natural grape juice fermentation by haploid wine strain C9 with and without 10 mg/L GA. Viable cell number were expressed as c.f.u/mL. Experiments were done in triplicate. The mean and standard deviation are provided. B) Spot assays on SD+uracil plates containing GA at different amounts of wine strains EC1118 and C9 and laboratory strains CEN.PK and BQS252 (*MAT* a *ura3-52*, a FY1679 derivative with S288c genetic background).

Supplementary Figure S2. Interaction between *GCN5* deletion and GA in winemaking conditions. A) Viable cell number in synthetic grape juice fermentations of the C9 wine strain and its *gcn2*D derivative with or without 10 mg/L GA, measured as cfu/mL. B) Survival curve along the aforementioned vinifications. Viable cell number at day 5 from Panel A was taken as 100% survival. C) Sugar consumption during fermentations. Experiments were done in triplicate. The mean and standard deviation are provided.

Supplementary Figure S3. Metabolomic data on the alteration of NAD salvage pathway by GA**.**

Supplementary Figure S1

A)

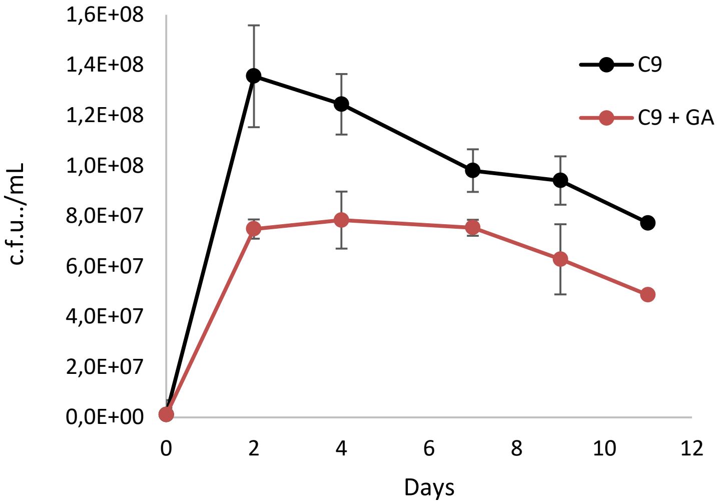

B)

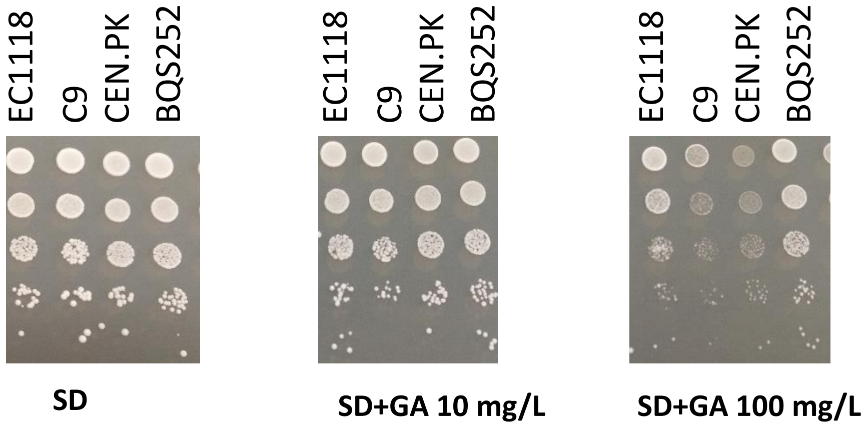

Supplementary Figure S2

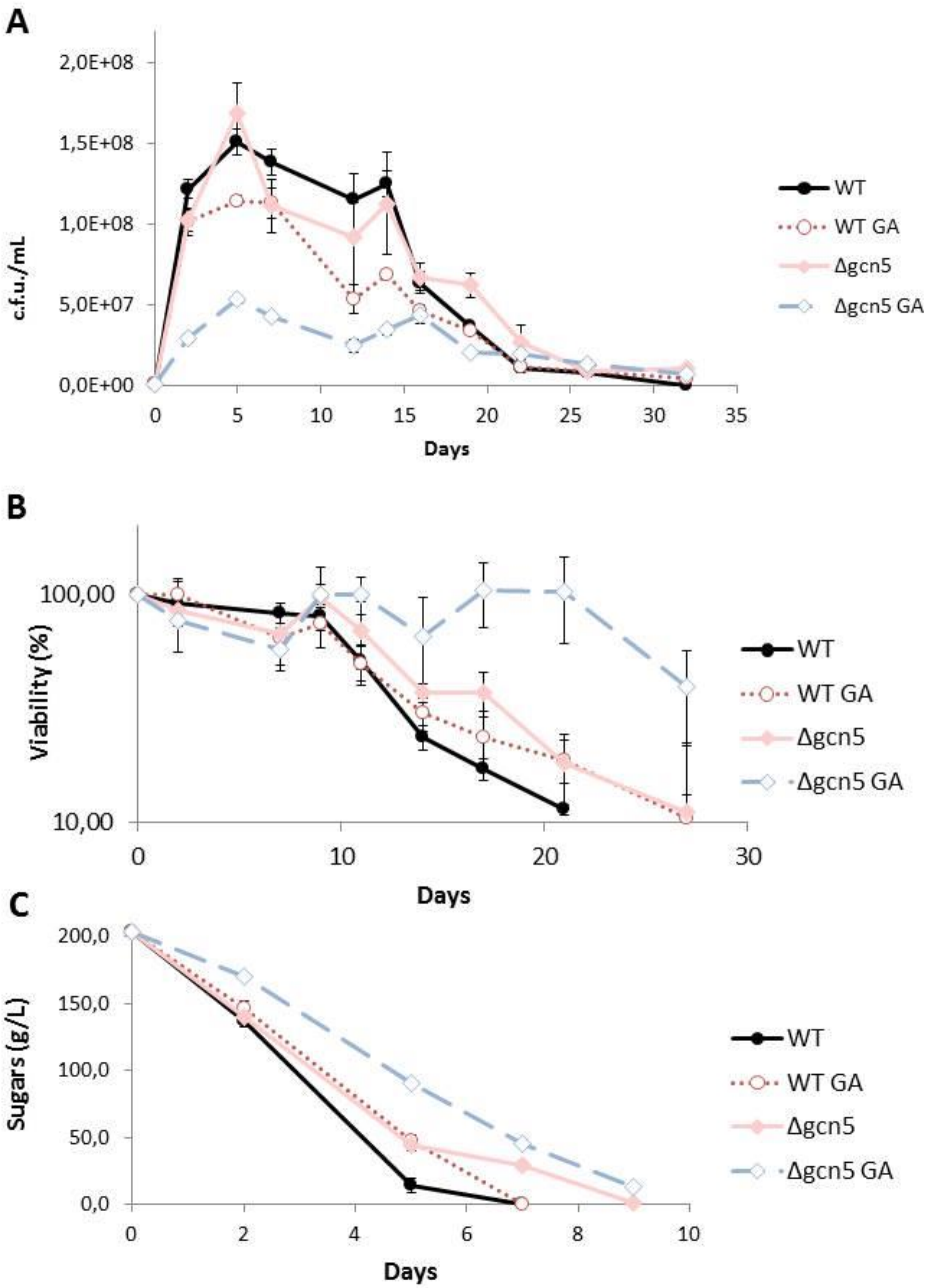

Supplementary Figure S3. Alteration of the NAD Salvage Pathway

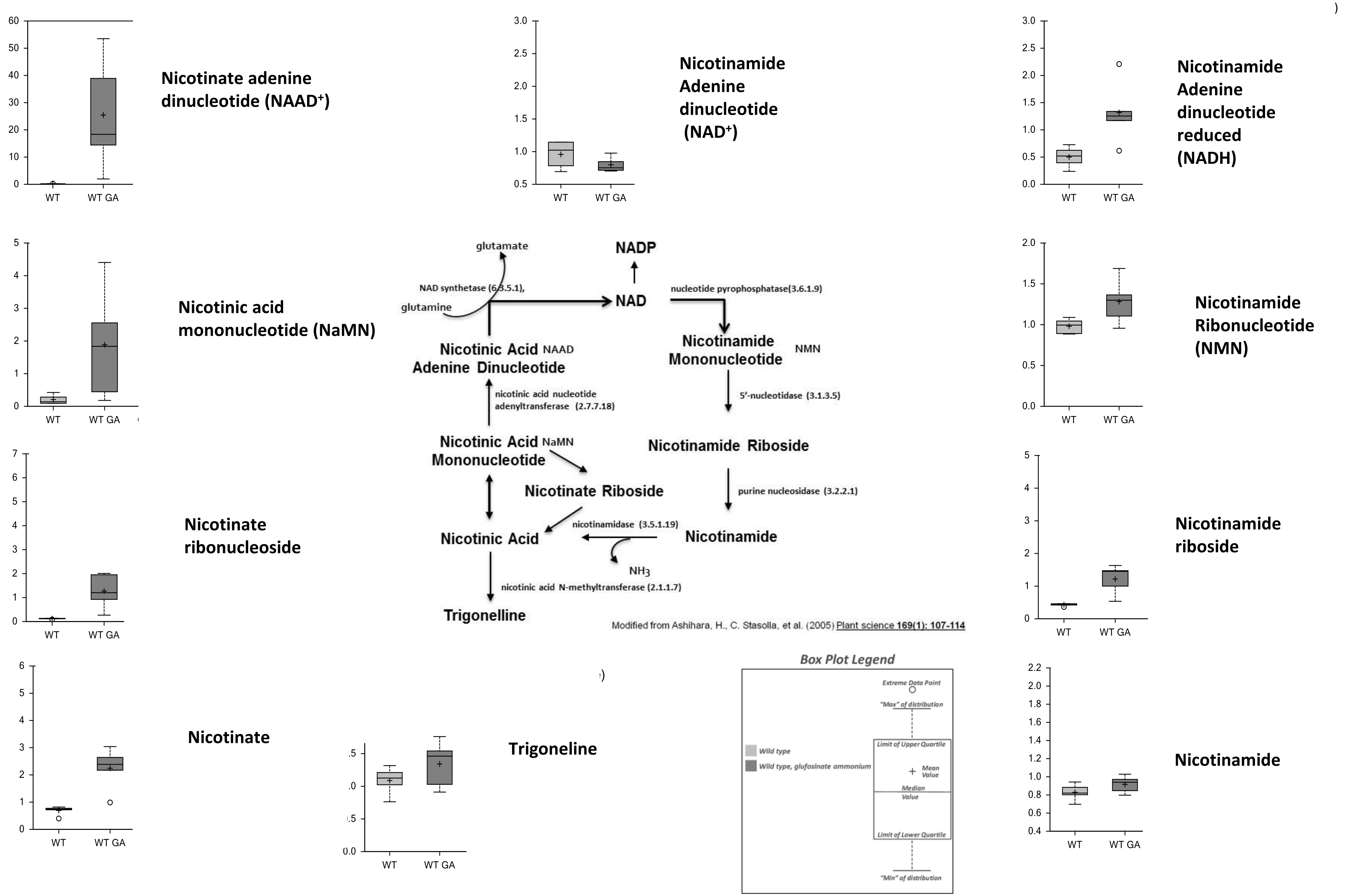

Original images from Figure 5 Western blot

p-Rps6

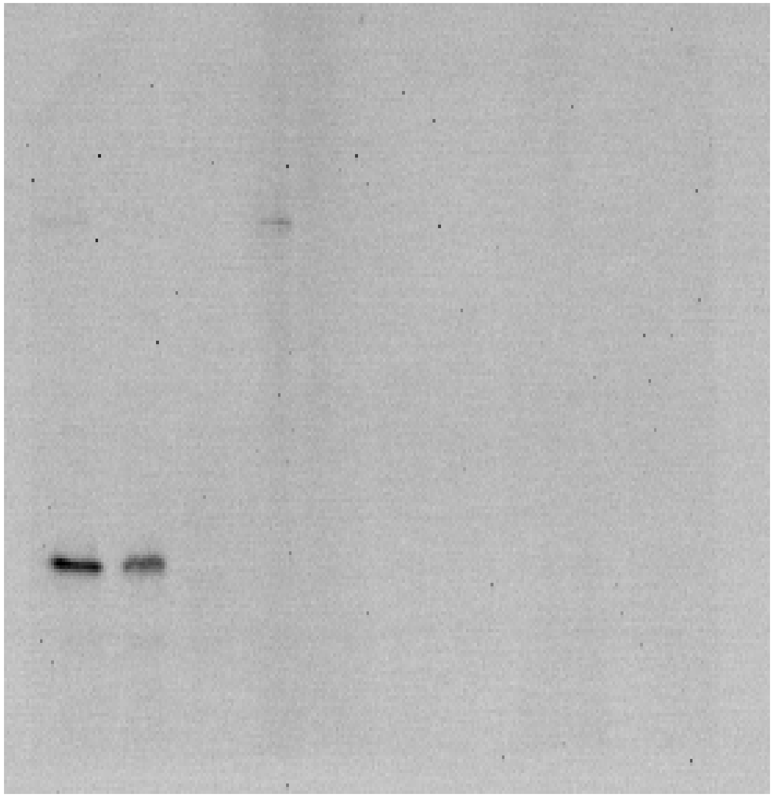

Rps6 loading control

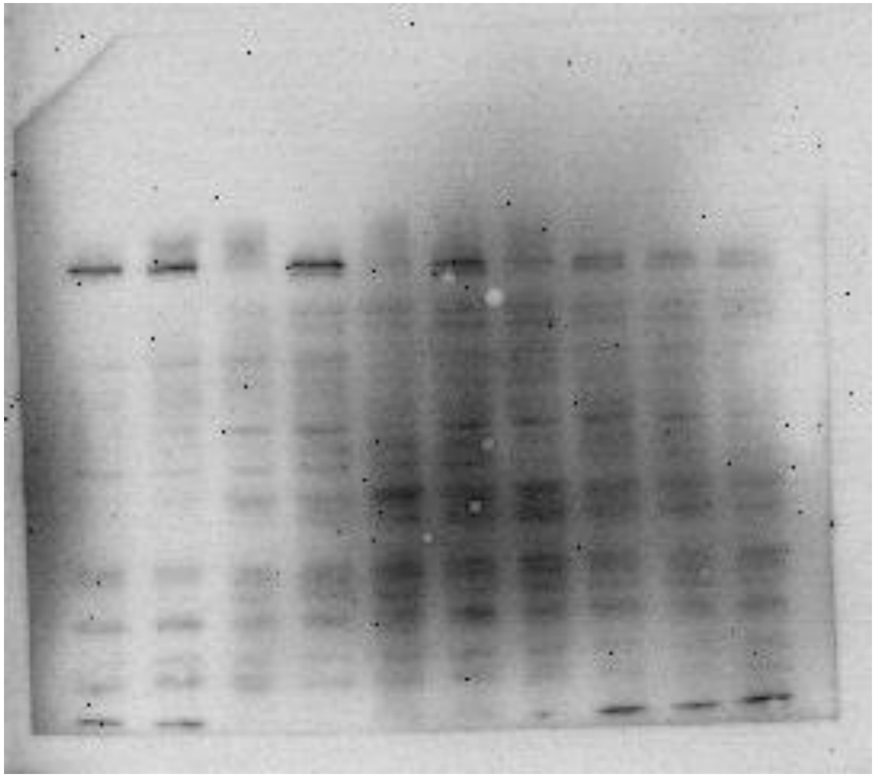

Supplement: Supplementary file 1 — Supplementary Information [file 41598_2017_12794_MOESM1_ESM.pdf]
